# Peer-assisted learning (PAL): skills lab tutors’ experiences and motivation

**DOI:** 10.1186/s12909-019-1760-2

**Published:** 2019-09-14

**Authors:** T. J. Bugaj, M. Blohm, C. Schmid, N. Koehl, J. Huber, D. Huhn, W. Herzog, M. Krautter, C. Nikendei

**Affiliations:** 1 Department of General Internal and Psychosomatic Medicine, University of Heidelberg Medical Hospital, Im Neuenheimer Feld 410, 69120 Heidelberg, Germany; 2 Department of Psychosomatic Medicine and Psychotherapy, Klinikum Esslingen, Esslingen, Germany; 3 Practice Drs./NL Albertus Arends, Heidelberg, Germany; 4 Department of Nephrology, University of Heidelberg, Heidelberg, Germany; 5 Clinic for Kidney, Hypertension and Autoimmune Diseases, Klinikum Stuttgart, Stuttgart, Germany

**Keywords:** Peer-assisted learning, Peer education, Peer teaching, Skills lab

## Abstract

**Background:**

Peer-assisted learning (PAL) is a common teaching and learning method in medical education worldwide. In the setting of skills laboratories (skills labs), student tutors are often employed as an equivalent alternative to faculty teachers. However, to the best of our knowledge, there is a lack of qualitative studies which explore the reasons for the personal commitment of student tutors. The aim of our study was to examine how undergraduate students experienced and evaluated their roles as skills lab student tutors, what their motivation was, and whether social and cognitive congruence played a role in their teaching experiences.

**Methods:**

We conducted in-depth, semi-structured interviews with student tutors who were currently teaching in a skills lab. After the interviews had been transcribed verbatim, two independent investigators performed a qualitative content analysis according to Mayring.

**Results:**

In total, we conducted nine interviews with student tutors. Our results revealed that all student tutors showed great enthusiasm and motivation for their jobs as peer teachers. One of the main motivating factors for student tutors to teach in a skills lab was the possibility to simultaneously share and improve their knowledge and expertise. In general, the participants of our study had high aspirations for their teaching. They found it particularly important to be empathetic with the student learners. At the same time, they thought they would personally benefit from their teaching activities and develop a certain expertise as student tutors.

**Conclusions:**

With the present study we are able to gain some insight into what motivates student tutors to teach in a skills lab and what kind of experiences they have. Our results provide an important input for the future training of highly qualified student tutors.

## Background

Peer-assisted learning (PAL) is an established teaching and learning method in medical education worldwide [1]. Most medical schools implement (near-)peer teaching programs by using students as teachers [2]. In pre-clinical medicine, PAL has been established in particular in anatomy [3–7], physiology [8, 9] and problem-based learning [10, 11]. In clinical medicine, this method is applied above all for the training of clinical skills, such as communication [12], physical examination [13–16], different technical procedures [17–22] and resuscitation skills [23, 24]. Furthermore, PAL is used to teach clinical procedures in specific programs for students who are currently doing clerkships or internships [25–28]. The concept of PAL even seems to be suitable in the area of stress prevention at medical school [29].

PAL is mutually beneficial both for student tutors and student learners [30–34]. On the one hand, PAL supports student learners’ cognitive, psychomotor and affective development. This results in an increase in self-confidence, autonomy, clinical reasoning, self-evaluation and peer collaboration [35]. On the other hand, student tutors also benefit from this program and are able to improve their individual knowledge, skills and attitudes, while practicing interaction and leadership competencies. This can enable student tutors to become better learners themselves, which in turn helps their undergraduate medical education, future residency, and faculty membership [30]. Some studies have shown that so-called ‘social and cognitive congruence’ between student tutors and student learners play an important role in PAL [2, 36–39]. As student tutors and student learners have similar social roles, it is assumed that they are ‘socially congruent’ [38]. This means that they share interpersonal qualities which facilitate informal and empathic communication as well as the establishment of a learning environment that encourages an open exchange of ideas and student learners’ personal concerns. The term ‘cognitive congruence’ refers to the assumption that student tutors and student learners are likely to share a similar knowledge base and similar learning experiences; they are on the same ‘wavelength’. This could mean that student tutors think of explanations that are more likely to meet the student learners’ needs and are therefore easier to understand [34, 36]. Thus, cognitive congruence could compensate for the lack of knowledge and expertise of peer-teachers compared to more experienced teaching staff [40, 41].

Teaching procedural technical skills in skills laboratories (skills labs) has become an indispensable part of medical education and is common in many medical schools [42]. Skills labs enable students to learn and practice specific procedures in a simulated and sheltered environment by using manikins, part-task-trainers, simulators, or simulated patients. In skills labs, it is quite common to have student tutors as teachers. Several studies have shown that the teaching methods of student tutors are either equivalent or even superior to those of more experienced teaching staff [20, 21, 34]. For instance, student tutors were able to create a more active learning environment compared to professors when teaching procedural skills [43]. However, to the best of our knowledge, there is a lack of qualitative studies assessing how student tutors evaluate their own teaching activities in a skills lab. Furthermore, it has not been investigated whether social and cognitive congruence are relevant factors in peer teaching. Only Weyrich et al. [44] qualitatively assessed whether peer teaching of technical skills for undergraduate students is feasible and accepted by both student tutors and student learners. This was investigated with a single open-ended question in their questionnaire.

Therefore, this study aimed to examine undergraduate students’ motivation to become student tutors in a skills lab, what they experienced while teaching and how they evaluated their experiences. Furthermore, we wanted to assess whether social and cognitive congruence were important factors. The aim of our research project is to make it easier for members of faculty to select student tutors and to develop a pool of tutors willing to teach in a skills lab. To explore the characteristics of the relationship between student tutors and their students in a skills lab, we conducted semi-structured interviews with nine student tutors.

## Methods

### Context

At the Medical Faculty of the University of Heidelberg, skills lab teaching was established in the year 2000 and has since been continuously developed and extended. All major clinical departments offer skills lab training sessions within their core curricula, either on a mandatory or voluntary basis. Lessons by faculty staff are complemented by peer-assisted learning – in most cases, the latter is voluntary. In addition, all final-year students in the Department of Internal Medicine are invited to participate in a specific skills curriculum to accompany their clinical placements [45, 46]. In general, third- to fifth-year medical students with some teaching experience are recruited as skills lab student tutors for the interdisciplinary longitudinal skills lab team on a voluntary basis [47]. However, skills courses and tutorials for final year medical students are held by medical doctors, as student-tutors should not teach more experienced peers. Skills lab student tutors receive financial compensation and are employed by the medical faculty as student assistants. The Medical Faculty of the University of Heidelberg offers regular tutor trainings to ensure appropriate teaching skills of their skills lab student tutors (see “Participants” below).

There is plenty of evidence that epistemological beliefs are an important component of student learning and that teachers’ beliefs affect their teaching as well as their pedagogical decisions [48–50]. Assuming that skills lab student tutors’ beliefs also affect students’ behavior and their clinical performance [51], the “educational environment” or epistemological grounding in the skills lab team will be described in a few sentences below. First of all, it has to be said that the skills lab is situated in a large department, the Department of Internal Medicine. Despite all efforts it cannot be ruled out with certainty that some teachers over-emphasize recall (rather than deeper thinking) and may not promote critical thinking skills. However, in the skills lab courses students are generally encouraged to work cooperatively which might create a suitable “environment” to help them develop the skills and beliefs needed to think critically [52]. In addition, more sophisticated epistemological beliefs seem to improve clinical reasoning and problem-solving competence [51]. Therefore student tutors are encouraged to give the students an idea of more “sophisticated” and advanced epistemological beliefs [53], such as

- knowledge is tentative and learning happens gradually,
- knowledge is acquired by the learner and not “given” by an authority,
- learning ability can be improved over time and individual effort.

### Study design

This is a descriptive, qualitative study. All participating skills lab peer-tutors were interviewed personally and individually according to a semi-structured interview guideline in the Department of General Internal Medicine and Psychosomatics, University of Heidelberg Medical Hospital, Germany. We chose a semi-structured interview (SSI) approach which is the most frequently applied interview technique in qualitative research [54] and in the health care context [55]. Evaluation of objective knowledge constitutes the framework for the development of the SSI guideline and the foci for the development of the SSI question stems. Because all participants are asked the same questions in the same order, data collected are comparable and helps the investigators to capture perceptions with regards to a specific situation or phenomenon. Therefore this method is especially useful when data is only collected through interviews [54] and when there is sufficient objective knowledge about a certain experience, but the subjective knowledge is deficient.

### Ethics

Study participation was voluntary. We informed all skills lab student tutors in detail about the study’s purpose and granted them anonymity and confidentiality regarding their data. Before starting with the interviews, we obtained written consent from all participants.

The Ethics Committee of the University of Heidelberg (No. S-423/2014) granted their approval. The study was conducted according to the Declaration of Helsinki (64th WMA General Assembly, Fortaleza, Brazil, October 2013).

### Participants

For recruitment we invited all ten skills lab peer-tutors (9 female, 1 male) who were currently teaching in skills labs as a part of the interdisciplinary longitudinal skills lab team [47, 56] of the University of Heidelberg, Germany, to participate in the study. Except for being a skills lab peer-tutor there were no further eligibility criteria for the inclusion of participants. As all skills lab peer-tutors were invited to participate (to capture even extreme views and attitudes within the sample) the current study did not use any additional exclusion criteria. Nine student tutors agreed to take part in semi-structured interviews. Written informed consent was obtained from all participants. We investigated their motivation for teaching, their perception regarding their teaching activities and the challenges they faced as tutors. To ensure the appropriate technical and didactic training of new tutors, the faculty offers regular tutor trainings which comprise three individual 3-h sessions led by consultants of internal medicine and a clinical psychologist [56, 57]. As a reference source, student tutors receive a comprehensive manual which contains a detailed checklist for every procedure they are taught during these tutor trainings [58]. Furthermore, they get the pocketbooks “Heidelberg standard examination” [59] and “Heidelberg standard procedures” [60] with accompanying online film material comprising faculty-wide standards for clinical physical examination and clinical procedures at the Medical Faculty of the University of Heidelberg. In addition, all student tutors are always welcome to observe other tutorials or participate in refresher courses.

### Questionnaire

A questionnaire asking about sociodemographic information, such as gender and age, and previous work experience (e.g. months of experience as a skills lab tutor, previous training and completed internships) was designed by the authors of this study (see Table 1).

### Interview guideline

Four skills lab experts, all actively involved in skills lab teaching and with a degree in medical education (e.g. MME), developed the study’s interview guideline. The guideline was based on a profound literature review, using a semi-structured guideline format [61–64]. The interview guidelines were developed in line with our general research questions: How do undergraduate students experience and evaluate their role as skills lab student tutors and what is their motivation for teaching? How relevant is social and cognitive congruence for their evaluation? The investigators developing the guidelines payed particular attention to use everyday language and avoid theoretical jargon in order to create a more natural atmosphere. In line with Helfferich [62], we asked the following key questions:

- (I) “What was your personal motivation and background that inspired you to work as a student tutor?”
- (II) “Could you please describe your teaching experiences as a student tutor in your own words?”
- (III) “How do you experience your relationship with the student learners?”
- (IV) “Could you please assess your personal impact on the general development of the student learners?”
- (V) “How was your personal development influenced by your role as a teacher in the skills lab?”
- (VI) “Could you please list some personality traits or characteristics that you feel are important for an “ideal” student tutor?

### Interview procedure

The interview procedure was in line with the COREQ (Consolidated criteria for reporting qualitative research) checklist [65]. Participation in our study was voluntary. We informed the skills lab student tutors about the background and goals of our study beforehand. Then we arranged individual appointments to interview the student tutors who had agreed to participate in our study. All interviews were conducted in person. At the beginning of the individual meetings, each student tutor was asked to fill in the aforementioned questionnaire asking about sociodemographic information and previous work experience (see Table 1). All interviews were conducted by a 30-year old male psychologist and research assistant at the University of Heidelberg who had been trained to conduct these interviews and was supervised by an experienced researcher. Each interview lasted approximately 30 min, followed the semi-structured interview guideline and was recorded on audio-tape.

### Qualitative content analysis and quantitative statistics

After verbatim transcription, all information that could identify an individual was removed from the transcripts. Next, the nine interviews were subjected to a qualitative content analysis according to Mayring, following the principles of inductive category development [66]. First, we undertook an open coding of all interviews (*n* = 9) to identify possible recurring topics. Next, individual sentences or passages were identified as one code, representing the most elementary unit of the resulting protocol [67]. Using the software MAXQDA (version 2010, VERBI Software - Consult - Social Research GmbH, Berlin), we summarized individual codes as relevant topics for each participant. Then, two independent analyzers compared recurring topics from the individual interviews and assigned them to higher-level categories. The analyzers discussed the respective codes and topics to reach consensus (investigator triangulation), or made adjustments, if necessary. Next, we subsumed the topics into a total of *n* = 7 relevant categories. The final coding framework included definitions for each of the categories. We applied the categories to all transcripts using the software MAXQDA. For the sample description, descriptive statistics were computed and are presented as means ± standard deviation.

## Results

### Participants

Nine skills lab student tutors (mean age 24.1; SD = 1.8) participated in the interviews (identified as P1 to P9); this is equivalent to 90% of the entire skills lab tutor team. They had a mean experience of 6.8 ± 3.4 months as skills lab tutors (see Table 1).

**Table 1:** Skills lab student-tutor base-line characteristics

Gender	Female = 8 (88.9%), male = 1 (11.1%)
Age	M = 24.1 (SD = 1.8)
Semester	M = 6.7 (SD = 1.8)
Subject	Medicine = 8
	Nursing education = 1
Months of experience as a skills lab tutor	M = 6.8 (SD = 3.4)
Prior areas of working as a skills lab tutor other than Internal Medicine	Pediatrics = 5
	Surgery = 1
	ENT = 1
Previous training	Paramedics = 1
	Health and nursing care = 1
	None = 7
Previous studies	Biomedical Sciences = 1
	Biology = 1
	Dentistry = 1
	None = 6
Completed internships^a^	M = 6.5 weeks (SD = 4.4)^b^
	Radiology = 1
	Orthopedics = 1
	Cardiology = 2
	Neurosurgery = 1
	Visceral Surgery = 1
	Internal medicine = 2
	Gastroenterology = 1
	Ophthalmology = 1

^a^not applicable for *n* = 1 student of Nursing education, ^b^duration of completed internships

### Main categories and topics derived from the qualitative analysis

We identified a total of 400 relevant single codes and seven main categories (with a total of 14 further subcategories). The main categories include (A) personal motives for working as a student tutor in a skills lab, (B) defining aspects of the relationship between student tutors and student learners, (C) practical aspects and experiences of working as a student tutor, (D) specific methodological and didactic approaches, (E) perceived expertise and dealing with classroom challenges, (F) self-expectation and self-reflection, and (G) personal development and the influence on how the student learners progress. In the following paragraphs, we will describe the seven main categories and give quotes from the interviews for further illustration. The number of citations which we included in the analysis of each main category is shown in parentheses. Please note that there will not be a separate “Discussion” section, as the study's results are instead discussed in immediate context of the citations.

#### Personal motives for working as a student tutor in a skills lab (12)

Many tutors described their strong motivation and enthusiasm for teaching as a key reason for their high level of commitment.

*“I really enjoy teaching other people (...) it gives me a good feeling that I can teach something to others and it’s a pleasure to see my students having fun [in the skills lab]” [P 3]*

Some of them stated that they had previous experience in private coaching or tutoring. The tutors found it particularly rewarding to be able to work closely together with students and share their knowledge with others. The majority of the student learners were perceived as open, interested, and motivated. They treated each other in a friendly and respectful manner.

*“I actually always look forward to (...) tutoring (...) because it is a good feeling - students who come want to learn something from me and I enjoy sharing my knowledge.” [P 5]*

In summary, two main motives for working as a student tutor in a skills lab could be detected: The improvement of professional skills and career opportunities (i) as well as a welcome opportunity for networking (ii).

##### Improvement of professional skills and career opportunities

The main motivational factors the interviewed student tutors gave were that they could share their expertise while also improving their own factual knowledge and procedural skills, while financial compensation seemed secondary. Indeed, students who are teaching other students in a domain that is relevant for their own career, do learn in a different way, possibly leading to a longer and more solid retention of knowledge [68]. Nevertheless, the provided financial incentive may have increased the tutors’ motivation to teach because working as a student assistant combines the ability to earn money with gaining professional experience that could lead to better career opportunities.

*“I liked the idea of becoming a student tutor because on the one hand, you learn something yourself – I mean, you need the procedural skills* [note from the authors: those trained in the skills lab] *yourself and you also learn to teach and all these other skills that you might need during your studies and on the other hand you meet other students* [...] *and of course there is also a financial incentive.” [P6]*

*“I find it positive* [...] *that you learn to teach and you really get to know the procedural skills* [note from the authors: those trained in the skills lab]*, so you improve your skills.” [P4]*

The citations make it very clear that the students were not only interested in improving their own set of medical skills (i.e. those skills taught in the skills lab), but were also hoping to acquire additional skills, such as teaching skills that are vital for all doctors, to gradually develop a “professional identity” [34]. This goes in line with a study by Burgess et al. which showed that PAL provided an opportunity for student tutors to practice and improve both, their medical knowledge and their teaching skills [69]. It is important to understand that teaching skills are more and more recognized as requisite graduate competencies [70].

##### Networking

Another motive the student tutors gave for their teaching activity was that they aspired to partake in networking with students and medical professionals.

*“I find teaching really exciting and I could imagine teaching a lot in my future life.* [ … ] *I would like to go to Pediatrics later on* [ … ]*. You get to know the doctors* [note from the authors: as a student tutor] *and I also found that a positive aspect.” [P8]*

Networking is an important aspect in medical education because learning and practice become inseparable when professionals work in communities of practice that create interpersonal bonds and promote collective learning [71, 72]. Communication skills are augmented by PAL [12, 73] and can be regarded as a key factor for effective networking. After all networking should be considered as part of the skills lab’s “hidden curriculum” [74] for both, students (who come in contact with senior students, i.e. student tutors) and student tutors (who come in contact with other members of faculty or other medical professionals). Actually, many student tutors stated that their initial interest in the job had been sparked when they had heard of the possibility to work as student tutors through friends or faculty members, which is a good example for the importance of networking even at this career stage.

#### Defining aspects of the relationship between student tutors and student learners (45)

Three aspects of the relationship between student tutors and student learners should be discussed: The tutors’ understanding of students’ learning needs (i), the special atmosphere of learning (ii) and the aspect of being a role model (iii).

##### Better understanding of students’ learning needs

The student tutors emphasized that their relationship with the student learners was at eye-level. They highlighted their wish to empathize with and understand the student learners. They felt able to relate to their students and shared their own experiences with studying medicine with them in an informal manner.

*“I think it's very important to meet your students at eye level (...) and not in a condescending way, along the lines of: “I know everything.” (...) I think it's important that (...) everyone finds it possible to ask any question whatsoever without feeling stupid.” [P 8]*

The student tutors stated that their close relationship with the student learners enabled them to assess and understand their level of knowledge which, in turn, facilitated finding individual solutions for particular difficulties. Some student tutors thought that more advanced medical teachers were less motivated in comparison. However, besides these differences to courses held by faculty staff, some student tutors sometimes found themselves taking on the role of professional lecturers. Others had more difficulties in accepting this role or reported not feeling like a professional teacher.

*“You kind of start to feel like a professional teacher, especially if you demonstrate the skill once and then explain the procedure once, you get into a teaching-role ( … ) and then you snap back to just being you. ( … ) That can feel a little strange sometimes, but it’s kind of an automatic process, you can’t really do much about it.” [P 9]*

The experiences that student tutors make strongly suggest that the concept of cognitive and social congruence is a relevant factor in near-peer teaching in skills lab settings [2, 37, 38]. This result can also be seen in a study by Yew and Yong (2014) which analyzed the qualitative feedback given by a large group of students regarding the importance of cognitively and socially congruent tutors [75] in problem-based learning. Furthermore, Schmidt and Moust [38] were able to demonstrate the importance of a congruent relationship between tutors and learners by showing that social congruence and successful group learning outcomes were linked together. In their model, social congruence and the used level of expertise have a direct, causal effect on cognitive congruence, the functioning of a small group and, ultimately, on academic success. Other studies investigating the effectiveness of PAL in skills labs have been able to show that PAL is as effective as or even superior to courses which are taught by faculty staff [20, 21, 34]. This underpins the idea that the combination of social and cognitive congruence makes PAL a very effective teaching method [36, 38].

##### Relaxed atmosphere of learning

The student tutors thought that their own enthusiasm and high motivation as well as their similar role and similar level of expertise were substantial factors for the positive and productive atmosphere in their sessions. The tutors found that students who were taught by their peers – rather than by more experienced teaching staff – were generally less anxious and felt fewer inhibitions in asking questions, talking about difficulties, and making mistakes. This goes in line with findings by Carr et al. [76] who also showed that PAL provided a comfortable learning environment where the students felt safe to ask questions or make mistakes. Nouh et al. were able to show (low) positive correlation between the perception about learning environment (measured via DREEM, i.e. the Dundee Ready Education Environment Measure) and medical students’ academic performance [77]. Consequently, one could argue that a relaxed learning environment, as provided by PAL, may enhance the academic performance of medical students. However, educational environment does not only affect academic performance: Enns et al. were able to demonstrate that educational environment also acts as an important moderator of medical students’ quality of life (QoL) [78]. Authoritarian teachers, overly teacher-centered teaching as well as angry teachers are some problems that could potentially counteract a relaxed educational environment [79].

*“We all know that we are much more willing to ask questions in a relaxed atmosphere. ( … ) I value this kind of atmosphere as a learner and I also try to create it when I work as a student tutor.” [P7]*

*“Yes, I'm trying to actively create a good atmosphere by being nice and friendly” [P1]*

##### Role model

Furthermore, the tutors thought that their own motivation and interest in the skills lab training sessions were crucial for their students’ motivation, contributions, and satisfaction. Some tutors saw themselves as a role model for students and noticed that their student learners observed, imitated (e.g. during role-play) and even admired their behavior. The tutors also observed that the students’ motivation and level of participation were decisively influenced by the student tutor’s approach to the lesson.

*“You usually show them the procedure once, and if you talk them through it properly, the students generally observe what you are doing and try to copy you.” [P 1]*

This seems to be consistent with fundamental assumptions of social learning theory which state that new behavior is learned through identification and imitation [80]. From what we know role models in medical education are of utmost importance, e.g. in the first year of medical school, where a transition from secondary education takes place which may create problems of socialization [68]. However, two of the nine interviewed tutors thought that some students preferred courses that were held by more experienced faculty members. In their opinion student learners were more respectful and appreciative towards more experienced teaching staff.

#### Practical aspects and experiences of working as a student tutor (126)

The student tutors discussed various practical aspects of teaching as well as specific experiences they had had during the courses. They described that they were patient with students and would invest a lot of time (sometimes even their free time after classes) to help them overcome difficulties or practice specific skills.

*“I'm rather calm. There was a situation in which students panicked before a test because they didn’t have time to practice with a member of the medical faculty (...) and then we practiced the skill again and again and I explained everything very slowly.” [P 7]*

Two main themes were discovered: The use of activities to elaborate on concepts (i) as well as the employment of open style of communication (ii):

##### Activities to elaborate on concepts

Regarding the student learners’ active involvement in the sessions, some of the student tutors reported that they would (a) ask questions or (b) give their students activities (such as group work) during the course. Other tutors stated that they would (c) invite their students to demonstrate a certain skill (e.g. using Peyton’s approach, see below) and (d) encourage them to ask questions. Some student tutors reported that they tried to (e) equally involve all members of their group in the activities. These activities are largely similar to those mentioned in other studies for the training of medical skills [81].

*“So, I always try to ask if anyone has any questions and sometimes I ask open-ended questions like “What would you do now?” (...). [P 6]*

*“And sometimes I also deliberately make mistakes to see if anyone notices; just to make sure that my students are paying attention.” [P 6]*

Most tutors stated to have benefited from the tutor training which had adequately prepared them for their teaching jobs (e.g. by teaching future student tutors all necessary skills to elaborate on concepts). Compared to sessions led by faculty staff, student tutors even considered their courses to be more structured. They felt that this also had an impact on their students’ exam preparation.

*“That’s the thing with student tutors, they’re specifically trained in [teaching the skills] exactly how [the examiners] would like to see them done in the exam” [P 3].*

##### Employ open style of communication

Most of the tutors made a particular point of the importance of empathizing with their students to gain a better understanding of their situation and their needs.

*“Sometimes, I also point out that I had problems learning a certain skill at first, too, or that it is normal to make mistakes in the beginning. I guess the most important thing is that you can give advice in an empathetic way.” [P 2]*

The tutors emphasized their efforts in creating a relaxed learning atmosphere in which mistakes were not seen as something bad. They tried to be kind and open towards their students without pressuring them or causing them stress. They hoped that this approach would encourage their students to ask questions without fearing humiliation.

*“...a good learning atmosphere without stress, pressure or being hectic” [P 4]*

The skills lab tutors stated that they would take an active stance towards disturbances (e.g. unmotivated students who did not want to participate or students who criticized other participants). Furthermore, they always tried to address problems openly and set clear boundaries. However, some student tutors found these aspects quite challenging.

*“If troublemakers are present (...) you really have to make sure you stay in control. I find that rather difficult.” [P 1]*

They further reported that they also tried to integrate communicative activities during model-based procedural skill demonstrations in order to practice “open style” physician-patient interactions, too. This is in line with other studies which showed that PAL can augment communication skills [73].

*“Which is why I think that practicing communication skills is also very important (...), for instance, to apologize and say: ‘sorry, unfortunately, I will have to do this again’, if you miss the vein with your needle, because I actually expect them [the students] to do the same with real patients.” [P 9]*

#### Specific methodological and didactic approaches (64)

The tutors described the methodological and didactic techniques which they used to structure their sessions and to explain the relevance of particular skills in clinical practice. In the following we will elaborate on the pedagogical methodologies and didactic approaches (i) with a special focus on Peyton’s approach (ii):

##### Pedagogical methodologies and didactic approaches

The participants of our study highlighted their efforts to actively involve all students in the lesson. They reported that they would apply interactive methods and didactic approaches during their courses and adapt them according to the student learners’ specific needs. The procedures or concepts they most frequently used were the Peyton’s approach (see below), which was typically blended with the first three steps of Gagné’s nine events (gaining attention, informing students of the objectives and stimulating recall of prior learning [82]), practicing communication skills, role play, giving active and constructive feedback, and model learning. This conceptual framework design, blending two acknowledged instructional design strategies, has been availed with success in other similar studies [83, 84]. In addition, many tutors mentioned that they make every effort to underline the clinical relevance of each and every skill (contextualization).

*“I try to point out to the [students] why the skill is important, (...) and where it is used in practice” [P 8]*

*“I think you can tell them about what you‘ve experienced with patients and give them [the students] examples like: ‘what kind of conflicts did I have with patients, how did I deal with them’, to give them real-life examples...” [P 7]*

Contrary to studies which report low acceptance among medical students concerning role plays [85], our results reveal that medical students being active as tutors (i.e. student tutors) regarded this as a good measure to teach communication skills and to make certain clinical situations more palpable. We may argue that role plays create a more realistic environment which enables students to actively engage in the lesson and therefore deepens their learning experience [86, 87]. Furthermore, role plays improve students’ communication with their peers [88]. Apart from actively engaging students in the lesson, a further important aspect for a successful outcome is the use of active feedback. Issenberg et al. [89] identified this as the single most important factor for effective simulation-based learning. Hattie further underlined the crucial role of active feedback by taking 800 meta-analyzes (comprising approximately 250 million learners) into account [90].

##### Peyton’s approach

In their study, Krautter and colleagues [91, 92] were able to prove the effectiveness of the Peyton approach to step-by-step skills training, which was mentioned by some of the interviewed student tutors as it was used for the demonstration and training of various procedural skills, such as drawing blood. This method was found to be superior to the approach of merely demonstrating specific skills. In addition, it seems to be highly conducive to simultaneously train communication skills and procedural skills – an aspect that seems quite straightforward given the fact that in practice both skills are inseparable. However, so far it seems to be common to regard these capabilities as separate and consequently teach them as independent skills [93].

*“ … you first demonstrate a skill* [note from the authors: first at normal speed, then with full instructions]*, next have them provide the instructions while you demonstrate the skill again, and finally they have to do it themselves.” [P5]*

#### Perceived expertise and dealing with classroom challenges (85)

Most student tutors predominantly perceived themselves to be competent and professional teachers and lecturers. They were confident in terms of their abilities and their classroom presence. However, some tutors felt insecure about their professional skills at the beginning of their teaching activity, especially when they were teaching students who were almost at the same level of their medical studies (e.g. due to a previous vocational training as a paramedic). Accordingly, the increase in self-confidence (i) and the emotional progression of student tutors (ii) will be discussed in more detail below:

##### Increase in self-confidence

The student tutors unanimously reported to have quickly gained confidence over time. In regard to their perceived expertise and competence, our results further suggest that work as student tutors increases the tutors’ sense of self-efficacy.

*“[I] actually think that I can convey the content pretty well and I would say that I am quite competent in the things that I teach.” [P 8]*

In line with our findings, a national survey of peer education revealed that college students who were working in a peer health education program noticed improvements in their knowledge on health, wellness and safety issues, in their teaching and public speaking skills, in their research and organizational abilities, and in their individual self-confidence, self-efficacy and self-understanding [94]. Interestingly, a study by Arrand suggested a greater improvement in personal skills for students with low self-esteem or confidence working as student tutors [95], whereas in academic reality, “high achievers” are often trained as tutors or mentors. Those students with low confidence might therefore gain more as student tutors and should not be ignored when hiring tutors.

##### Emotional progression

One tutor described the inner emotional progression over the course of a session to illustrate the demands of teaching: In the beginning the tutor would feel an initial state of apprehension and would become more and more relaxed during the class if everything went as planned. Another student tutor stated that it could be hard to remain self-confident when the students’ questions went beyond the specified course content. This was particularly difficult when questions addressed topics that the student tutors felt they lacked experience in or did not have enough knowledge of on.

*“It is not so easy to teach others something which you yourself haven’t done more than one or two times before - especially when you haven’t got that much practical experience yet, either. For instance, we practice inserting a gastric tube. I did that once, but I would never claim that I can do this very well. (...) It is a different story when it comes to drawing blood and suturing, which is easier to explain as I have more experience there myself.” [P 9]*

However; the majority of the student tutors said that they would calmly point out their own gaps in knowledge or address the course’s specific learning objectives. Further challenges in the classroom were unmotivated students, exchange students with insufficient language skills, and very heterogeneous groups in terms of competencies and knowledge. The student tutors discussed the need to overcome personal reserve in order to teach a particular skill, e.g. when they did not entirely conform to specific aspects of the syllabus they were expected to teach. Additionally, the tutors felt challenged to think of an adequate structure and pace for a lesson as well as keeping organizational aspects in mind.

*“Well, I think it’s very important to have a clear structure (...) and to be able to communicate some kind of structure. You shouldn’t just talk all the time without coming to the point of things. The students get bored quickly and you have to have a clear structure to your lessons.” [P 3]*

In line with these findings narrative inquiries by Arrand highlighted that student tutors may struggle with how to act in difficult situations, not always knowing how to manage the complex (and often inter-personal) demands in peer tutoring [95]. In summary, self-confidence and professionalism are needed for teaching peers in a skills lab, but the student tutors also described how important it was to have a clear structure, manage time efficiently, and meet organizational demands. Thus, tutors require leadership skills to manage lessons effectively and instill sufficient discipline to ensure an adequate learning environment for all students. At the same time, leadership skills are an integral part to medical practice which means that acquiring these abilities will also serve student tutors in their future careers. Unsurprisingly, leadership skills have been identified as an important competence within the CanMEDS roles which a responsible physician requires in a complex health care system [96]. Thus, it would be crucial to pay more attention to the development and regular practice of such competencies in modern tutor training programs [97, 98].

#### Self-expectation and self-reflection (35)

The interviewed student tutors also elaborated on specific qualities an ideal student tutor should have (i) and on the importance of self-reflection (ii):

##### Qualities of a good skills lab peer-tutor

In their efforts to provide successful skills training, all student tutors aspired to be perceived as confident and competent and endeavored to make the sessions as interesting and appealing as possible. They listed the following specific qualities that a good tutor should have:

- professional competence/ content-related knowledge and expertise in the medical subject of the skills course (e.g. ECG recording or taking blood samples)
- didactic skills in terms of having skills to plan, implement and evaluate skills lab teaching as well as to use a variety of teaching and assessment methods
- pedagogical competence, i.e. having basic educational knowledge to describe, analyze, evaluate and justify one’s own teaching practice
- organizational skills, which can be understood as the ability to make use of one’s time, energy and resources in an effective manner, e.g. by meeting targets and working independently
- personal motivation/ intrinsic motivation to work towards becoming a good tutor

Further, they considered a friendly, open manner and a quiet, patient, and empathic demeanor as favorable, as well as enthusiasm for teaching, authenticity and approachability. Being able to relate to the student learners, showing genuine concern for their needs, and treating them at eye level were also mentioned as important characteristics. Lastly, student tutors thought that a loud speaking voice was advantageous.

*“[A tutor] should be quite knowledgeable but should not necessarily just deal with the small print. [Tutors] should be able to focus on the essentials, too, have patience, be open, enjoy teaching other students, and have a very high degree of dedication and reliability.” [P 4]*

These aspects are largely in line with Sutkin et al.’s [99] description of a “good clinical teacher in medicine”: The study characterizes an excellent clinical teacher as being inspiring and supportive, as well as actively involving students in the lessons and communicating with them. The Association for Medical Education in Europe (AMEE) identified similar characteristics and used these aspects to define twelve roles (e.g. on-the-job role model, student assessor, clinical or practical teacher) into which ‘good’ teachers step when holding a course [100]. Nevertheless, the question remains how students with the potential to become good tutors can be identified at an early stage. So far, there are several factors that play a role in the selection of suitable candidates for tutoring jobs: a personal interview conducted by an experienced medical teacher, previous tutoring, teaching or leadership experience, completed internships or clinical placements, previous communication training, good grades in the relevant subjects, personal motivation [32, 44, 101].

Contrary to these criteria, Sobral [32] was able to demonstrate that grades had little predictive validity with regard to students’ tutoring qualities. Wadoodi and Crosby [102] even explicitly advise against selecting tutors according to previous academic achievement. The authors argue that weaker students will be more apt at identifying their students’ needs. Instead, the student tutors’ extent of content-related knowledge and expertise in the relevant skills should be of paramount importance. This aspect is underlined by a study by Rogers et al. [103] who demonstrated that peer teaching of surgical skills can in fact have a negative impact on the training outcome. Thus, even when students who seem suitable to become student tutors have been added to a pool of candidates, each teaching job would require that a good teacher is picked according to the individual course format. Here, the aforementioned [100] teaching roles that can be identified with a self-assessment survey could be an effective means to identify suitable teachers for individual courses. Future research projects should assess whether a modification of this approach is feasible in the field of peer tutorials. For example, tutors could keep portfolios listing their personal development and self-assessing their progress with regard to individual roles (such as ‘facilitator’, ‘role model’, and ‘information provider’). This could be accompanied by regular supervising discussions with the medical director of the skills lab. In order to ensure that these efforts are worthwhile, there would have to be other specific measures to develop the individual student tutors’ ‘role portfolios’, such as specific courses.

##### Self-reflection

The student tutors were very aware of their responsible role and they reported that they would frequently reflect their work critically to accommodate the students’ needs and suggestions.

*“And from the questions the students ask you, you can always learn how you could become better – which aspects you should put more emphasis on in the future; it’s important to remain aware of that and try to improve – students always give you good feedback for that.” [P1]*

The student tutors mostly referred to reflection on action, which can be considered as retrospective contemplation of practice (i.e. looking back after the event has occurred) [104] whilst reflection in action is happening during the event. The focus of reflection on action lies on self-development as information is turned into knowledge. Reflection on action might ideally lead to a practice of reflection before action (i.e. planning before acting), which can be observed in more experienced student tutors. Some evidence exists that peer-tutoring increases the capacity to reflect for the student tutors [94, 105].

#### Personal development and the influence on how the student learners progress (33)

The study participants indicated that their work as student tutors helped them to improve their confidence and develop their technical (i.e. medical skills like taking blood samples) and didactic skills (i.e. skills to plan, implement and evaluate skills lab teaching). Furthermore, they felt that they were able to approach the student learners with a more open, tolerant, and patient attitude as their teaching experience grew. One tutor thought that he/she had developed an improved sense of responsibility and was now able to show more initiative in other situations because of the role as a teacher.

*“I have not been here very long, but I would say that my self-confidence and my general confidence in my own practical skills have improved most.” [P 2]*

Due to the briefness of time they spent together, the student tutors stated that they only had a limited influence on their students in general, and on the students’ personality in particular. However, they stated that some students might be inspired by them and that the students’ positive experiences during class might spark their interest in clinical activities or tutoring. In addition, tutors felt that student learners might have gained a new perspective on physician-patient communication as well as increased their sense of self-awareness and self-efficacy.

*“I usually only see them [the students] once and for no more than 1.5 hours. That is such a short period of time that I doubt that I make a lasting impression. (...) Well, maybe they have gained self-confidence because they were able to learn some skills right away, and that's always encouraging, isn’t it?” [P 9]*

### Limitations

Although undergraduate medical student tutors teach in skills labs all over the world, the present study is, to the best of our knowledge, the first to explore their motivation for this job, their experiences and evaluation of their role and the relevance of social and cognitive congruence. Closing this gap with our results could have implications at an institutional level for the training of staff working in skills labs. However, our study has some limitations. Firstly, the results of the present study and therefore our findings’ generalizability are limited by the small number of participants due to the qualitative approach which is based on in-depth interviews and their analysis. However, it is important to point out that in qualitative research data saturation is not necessarily related to the number of participants; rather, the depth of the generated data has to be taken into account. Due to the fact that we conducted very detailed interviews, we were able to obtain a lot of data - despite the low number of study participants. After all, rather than being predetermined by statistical power analysis, qualitative research samples are very much dependent on the nature of the data [106] and in this case the availability of participants, i.e. the total amount of student tutors. Secondly, qualitative methods are generally more susceptible to a certain bias. This means that the interviewer may have influenced the study participants’ answers in the interviews and therefore may also have altered the outcome of our study (i.e. its validity which expresses the degree to which a measurement measures what it is supposed to measure). We tried to counteract this by employing an experienced interviewer who neither had any private or professional connection to the skills lab, nor the individual student tutors. The interviewer received feedback and support from other experienced colleagues on a regular basis (e.g. to avoid misperceptions with regard to what the interviewee is saying or with regard to what is being asked [107]). In addition, four skills lab experts were involved in developing the study’s interview guideline, which was based on a profound literature review. The sum of these actions might have led to higher validity by minimizing the possibility of bias [107]. Finally, it has to be mentioned that we only studied the experiences and impressions of the student tutors without interviewing the student learners. Thus no feedback on the student tutors’ performance was collected.

## Conclusion

The present study gives an insight into student tutors’ experiences of peer teaching. This helps faculty members gain a better understanding of student tutors’ motives for teaching. Furthermore, the present study shows that the theoretical concepts of social and cognitive congruence are indeed of high practical relevance for student tutors in a skills lab. The interviewed tutors pointed out that social and cognitive congruence with student learners enabled them to interact at matched levels, which in turn increased their enthusiasm for teaching, and was one of the main reasons for their personal commitment. In addition, the tutors listed other relevant motives for working in a skills lab, such as being able to share their expertise with others while improving their own factual knowledge, procedural skills and teaching abilities. Financial compensation only seemed to play a minor role. Our insight into these personal experiences could also have implications for the development of skills lab staff training at institutional level. Medical faculties might be able to improve their selection of students who are suited to tutoring jobs and even develop a pool of available tutors. However, further research is needed to identify, select and train the ‘ideal’ student tutors.

## Data Availability

The datasets used and analysed during the current study are available from the corresponding author on reasonable request.

## Abbreviations

AMEE: Association for Medical Education in Europe
COREQ: Consolidated criteria for reporting qualitative research
DREEM: Dundee Ready Education Environment Measure
ENT: Ear, Nose & Throat
M: Mean
MME: Master of Medical Education
PAL: Peer-assisted learning
QoL: Quality of life
SD: Standard deviation
SSI: Semi-structured interview
WMA: World Medical Association
